# Statistical analysis of dendritic spine distributions in rat hippocampal cultures

**DOI:** 10.1186/1471-2105-14-287

**Published:** 2013-10-02

**Authors:** Aruna Jammalamadaka, Sourav Banerjee, Bangalore S Manjunath, Kenneth S Kosik

**Affiliations:** 1 Department of Electrical and Computer Engineering, University of California Santa Barbara, Santa Barbara, CA, USA; 2 Department of Molecular and Cellular Neurobiology, University of California Santa Barbara, Santa Barbara, CA, USA

**Keywords:** Dendritic spines, Rat hippocampal culture, Linear network K-function, Morphological modeling, Spatial statistics, Point processes, Neuronal growth

## Abstract

**Background:**

Dendritic spines serve as key computational structures in brain plasticity. Much remains to be learned about their spatial and temporal distribution among neurons. Our aim in this study was to perform exploratory analyses based on the population distributions of dendritic spines with regard to their morphological characteristics and period of growth in dissociated hippocampal neurons. We fit a log-linear model to the contingency table of spine features such as spine type and distance from the soma to first determine which features were important in modeling the spines, as well as the relationships between such features. A multinomial logistic regression was then used to predict the spine types using the features suggested by the log-linear model, along with neighboring spine information. Finally, an important variant of Ripley’s K-function applicable to linear networks was used to study the spatial distribution of spines along dendrites.

**Results:**

Our study indicated that in the culture system, (i) dendritic spine densities were "completely spatially random", (ii) spine type and distance from the soma were independent quantities, and most importantly, (iii) spines had a tendency to cluster with other spines of the same type.

**Conclusions:**

Although these results may vary with other systems, our primary contribution is the set of statistical tools for morphological modeling of spines which can be used to assess neuronal cultures following gene manipulation such as RNAi, and to study induced pluripotent stem cells differentiated to neurons.

## Background

Spines are protrusions that occur on the dendrites of most mammalian neurons. They contain the post-synaptic apparatus and have a role in learning and memory storage. Spine distribution is a critically important question for multiple reasons. Changes in spine distributions and shape have been linked to neurological disorders such as Fragile X syndrome [1]. Spine distributions determine the extent to which the neuropil will be electrically sampled, i.e. dense distributions will sample the neural connectivity map more fully [2]. Furthermore, the nature of optimal sampling is unknown and likely depends on the surrounding anatomy and the total information content available to dendrites. Because pruning takes place during development in an activity dependent manner, spine distributions may reflect activity within neural circuits. Distributions of spine types are biologically important because the electrical properties of spines, such as the spine neck resistance, promote nonlinear dendritic processing and associated forms of plasticity and storage [3] to enhance the computational capabilities of neurons.

The shapes and types of dendritic spines contribute to synaptic plasticity. Because neighboring spines on the same short segment of dendrite can express a full range of structural dimensions, individual spines might act as separate computational units [4]. Nevertheless, the dendrite acts in a coordinated manner and thus the spatio-temporal distributions of different spine types is likely to be significant. Little is known about this population level organization of dendritic spines. Our aim was to perform an exploratory analysis of neuronal data from different time periods during the growth of rat dissociated hippocampal neurons, a well-established model system [5]. The observations here pertain only to the culture system and not necessarily to in vivo settings although the analytical tools used here could be adapted to in vivo analyses.

By quantifying populations of dendritic spines with automated tools at a global level, we were able to perform a much larger and more comprehensive analysis than most previous studies. Many studies only analyze a small region of interest on the largest dendrites, for example the 50–75 *μm* closest to the soma [6], or 10 *μm* segments [7], making it easier to measure manually the spine type counts and dimensions. Other works determine spine lengths and widths by manually drawing a line along the maximal length and measuring the length of that line [8], and therefore are only able to analyze a few neurons and a few hundred spines at a time.

In this study we determined the ratios of spine types along the dendrites as a function of time in culture, clustering or repulsion of spines in space, and how best to model spine type distributions. A model that fits the spatial distribution of spine types in healthy cultured neurons would be useful to assess neuronal cultures following gene manipulation such as RNAi and to study features of induced pluripotent stem cells differentiated to neurons.

Log-Linear Models (LLM) and Multinomial Logistic Regressions (MLR) are two basic and essential statistical methods, and have an extensive history of being used in biological studies. However, these tools have not been used thus far in the analysis of spine distributions. We fit a log-linear model (LLM) to the contingency table of spine features to determine the dependence between spine types (mushroom, thin, and stubby), distance from the cell body along the dendrite (in micrometers), the branch order of the dendritic segment on which it lies (primary, secondary, tertiary, etc.), and the day in vitro (DIV) on which it was imaged. Once we determined which of these attributes contributed to the overall dendritic spine model, we then asked whether these attributes can predict the occurrence of spines and of spine types. To answer this question we used a Multinomial Logistic Regression (MLR) model, which predicted the spine type, using the attributes that were found to be important through the LLM and associated contingency tables.

Finally, to understand how the dendritic spine density varied over the length of the neuron or whether the appearance of spines was completely spatially random i.e uniformly distributed over the neurites, we made use of spatial point processes. Spatial point processes have been used before in biological studies to model the locations of entire neurons [9-11], locations of ants nests [12] or xylem conduits [13]. There have also been other more ad-hoc methods created to study the number of "clustered spines" on each dendritic segment in monkey brains, where a cluster is defined as a group of 3 or more spines [14] however we believe our use of the linear network K-function [15] is the first work to analyze the locations of dendritic spines and their clustering properties in such a principled manner. Our analysis indicated that the density of spines is generally completely spatially random (CSR) over the dendritic length probably due to the absence of instructive directional signals found an in vivo setting in which spine distributions are unlikely to be CSR.

## Methods

### Cell imaging

Dissociated hippocampal neurons from embryonic rat brains (E18) were plated onto poly-l-lysine coated coverslips. Once neurons adhered to the coverslip, they were placed face-down on glial cells grown in vitro for 15 days. These neurons were a primary neuronal culture system, and no cell line was used. Neurons were grown for specific time periods up to 21 days in a neuronal medium containing B27. This co-culture of neurons and glia mimic the physiological conditions of neuronal growth and development in mammalian brain [5]. Work with the neuronal cultures was approved by the UCSB animal care committee.

To fill the neuronal processes including dendritic spines Green Fluorescent Protein (GFP) was expressed from a plasmid containing the beta-actin promoter (CAG-GFP) [16]. Of this plasmid, 2 *μg* was transfected into each coverslip containing about 50,000 neurons (including about 20% glial cells). Transfection was performed as described in the manufacturer’s protocol (Lipofectamine 2000 from Invitrogen) with minor changes. The transfection mix and neurons were incubated for two hours to avoid toxicity caused by lipo2000. Following transfection, coverslips were flipped back onto the glial dish, where they were originally cultured. GFP-actin transfected into the neurons at DIV4 (Day In Vitro) and neurons were studied at three time points- DIV7, 14 and 21. These time points survey the maturation period over which synapses and spines emerge [17]. Note that these were not the same neurons studied over time, but each time point represents a different population of neurons which were grown in culture up until the point of imaging. In this way our analysis represents a study at the population level. At each time point the number of images taken per plate depended on the transfection efficiency of that plate. On average approximately 1% of cells were transfected. The plating density was set so that neurons were relatively isolated in order to capture one neuron per image. An Olympus FluoView laser scanning confocal microscope was used. Image slices were 2048 by 2048 pixels at 154*nm* per pixel resolution. There were 7–33 z-slices per stack depending on the depth of the neuron, taken at 200*nm* steps. This means that the stacks were 315.39 *μm* × 315.39 *μm* × 1.4–6.6 *μm*. The z dimension slices were used to capture each depth level at the optimal focus, however we cannot claim to have accurate volumetric information at this resolution. A 40*X* oil objective lens with no optical zoom was used. Numerical Aperture (NA) was 1.3, and illumination conditions were kept constant. Deconvolution of the raw data before processing was not necessary because the images were clear enough to manually annotate the neuron traces and manually edit all the spine detections and types as described in the following section. We performed three biological replicates, the results of which are detailed below.

Although there are other higher resolution, full volume methods, the analysis of this data is broadly applicable to imaged neurons in other systems [5]. We attempted to capture the entire neuron in each image, however because of limits in available imaging techniques we found that this does not always happen. In the cases where dendrites were truncated at the end of the image plane we assumed that the proportion of spines in the missing data was similar to what had already been observed, and therefore the resulting distributions did not change. We verified this assumption visually by taking tiled mosaics of a few neurons imaged in their entirety from each DIV and checking that the branch orders, distances to soma and spine type counts were unchanged as compared to those of the same DIV. There was an observed increase in the dendritic length truncated by the image plane as the DIV increased. However in our particular analyses the methods used, such as the Log-Linear Model and Multinomial Logistic Regression, were focused on trends between spine characteristics such as distance to soma and type and these trends are innately unaffected by the truncation of dendrites given the above assumption. In addition, spatial point process analyses such as the linear network K-function always include the specification of an observation window [18], which in our case was the image plane. We verified (see Results and discussion section) that the overall spine density and the density of each spine type did not vary with distance from the soma so that we could assume spine density at the ends of the dendrites which were truncated was similar to the dendritic length which was observed. We recognize that we cannot see the proximity of labeled cells to other neurons which haven’t taken up the GFP labeling. These unlabeled neighboring neurons may cause some difference in spine distributions which we cannot quantify. For this reason we have attempted to quantify our biological findings statistically over entire experiments and DIV time points instead of by individual neurons, although in certain cases showing results from individual randomly sampled neurons was necessary.

### Neuronal reconstruction

There exist many automated methods for studying neuronal growth and morphometry and therefore we present a brief review of available software for tracing dendrites and detecting and classifying spines. In particular, NeuronJ [19] is a widely used software; however it is only semi- automatic and one must click several points to trace each neurite. The labeling is done manually and the statistics output only include lengths of neurites and not spine data. HCA-Vision [20] is a costly software with similar goals, however the parameters of the neurite tracing are set manually with a sliding bar and thus results require much hand-tuning. In addition, it is also focused on tracing neurites as opposed to spine analysis. For a full review of existing methods and softwares for neuron tracing and spine detection see [21]. We found NeuronStudio [22-24] to be the most user-friendly, and for this reason we used it to annotate dendrites and spines for this analysis.

**Figure 1:**
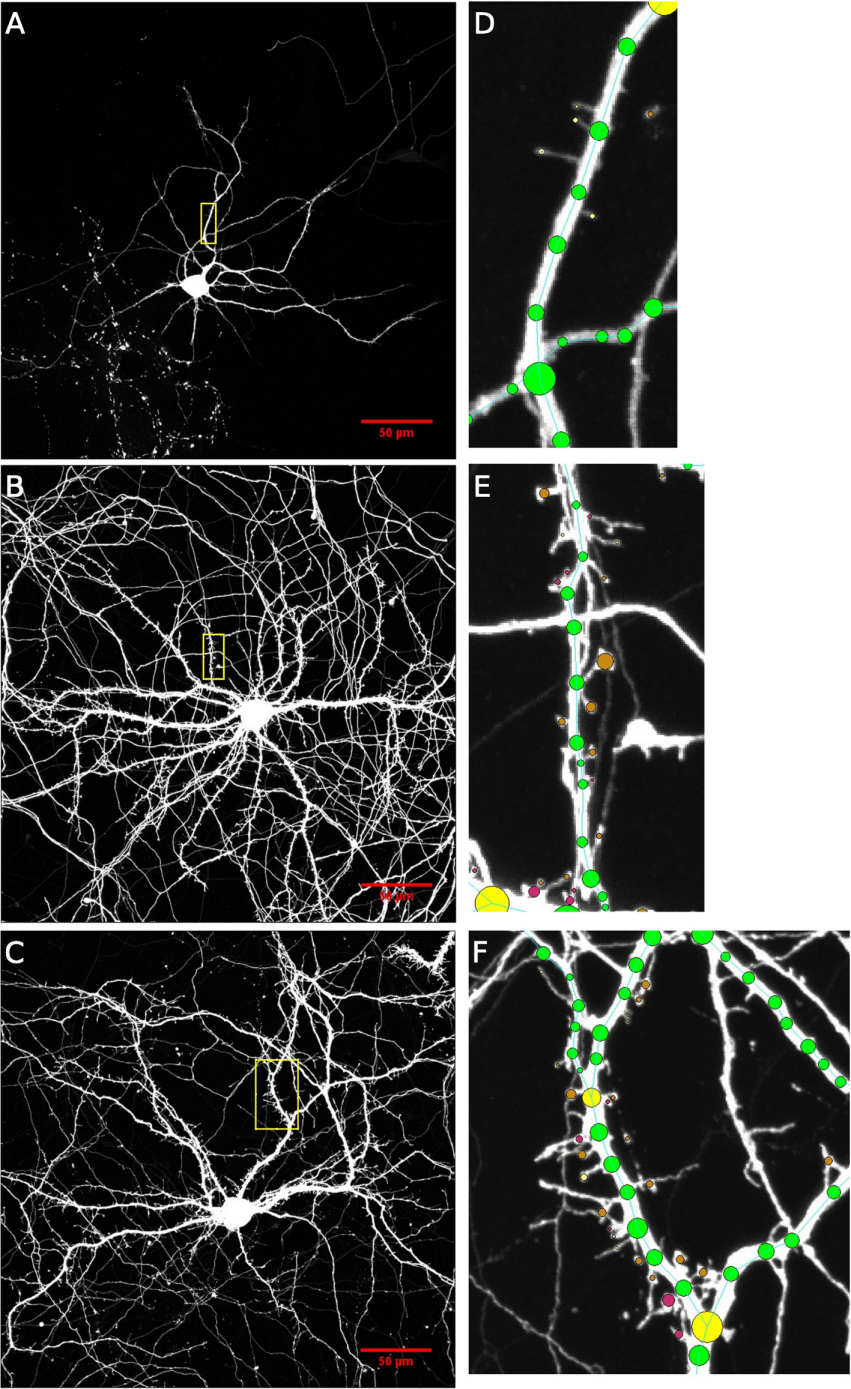
**Examples of cell imaging results.** This figure shows example images from each DIV (in order from top to bottom: DIV7, DIV14, DIV21) along with corresponding close-up images of dendritic segments where spines were clearly visible. Scale bars are shown in red in panels **A-C** and the yellow rectangular boxes in panels **A-C** show the region of interest which has been zoomed in on in panels **D-F** respectively. Panels **D-F** are all at the same resolution.

Despite the abundance of automated softwares, neuronal reconstructions are still largely performed by hand [25] and this is is especially essential for a study like this one, where the traversed distance of the dendrites and number of spines and their shapes were analyzed in such detail. Using automated reconstruction algorithms on raw data is prone to both false positive and false negative detections of spines, as well as misleading spine shape measurements. In cases where neurites from neighboring neurons enter into an image (e.g. Figure 1 panes B and C), NeuronStudio often incorrectly traces these neurites as belonging to the neuron of interest. For this reason we manually traced each dendritic branch and soma of each neuron, ran NeuronStudio’s automated spine detection/classification algorithm and then manually inspected and verified each spine’s location and type. The verification and tracing were done by the primary author and an undergraduate biology student working in the Kosik Lab (see Acknowledgements). They were both familiar with dendrite and spine morphology and the resulting annotations from each were cross-checked by the other.

Relevant spine attributes output from the NeuronStudio software include branch order (BO), type (stubby, mushroom or thin), distance to soma along dendrite (SD), length (tip of spine to dendrite) and width at widest point (head diameter or HD). However since NeuronStudio uses the length and width of the spines to determine the spine type, we chose to make use of spine type and discard the other 2 measurements. NeuronStudio uses centrifugal labeling for branch orders, meaning it starts at 1 at the cell body and moves outwards, incrementing at every y-shaped bifurcation regardless of the diameter of the daughter branches. Note that the entire image stack with z-dimension information was loaded into NeuronStudio for the spine classification, and that the software has interpolation algorithms to estimate the spine type in 3D. For spine detection the default cut-offs were used, i.e. a required spine height between 0.2–3 *μm*, a maximum spine width of 3 *μm*, a minimum stubby size of 10 voxels (at the imaging resolution given above), a minimum non-stubby size of 5 voxels, and automatic z-smear compensation. For spine classification, the default settings were also used, i.e. a head-to-neck ratio threshold of 1.1 *μm*, an aspect ratio (spine height-to-width) threshold of 2.5 *μm* and a minimum mushroom head size of 0.35 *μm*. NeuronStudio delineates spine types by these 3 thresholds. It is generally known that mushroom spines have a large head and a narrow neck, thin spines have a small head and a narrow neck, and stubby spines display no obvious subdivision in head and neck. If the head-to-neck ratio is above the threshold and the minimum mushroom head size is met, the spine is considered mushroom. If both the head-to-neck and aspect ratios are lower than the respective thresholds then the spine is considered stubby. The remaining cases result in thin spines. For further information on NeuronStudio reconstruction, detection, and spine classification algorithms please refer to [22,23]. In addition to the spine information, a trace file is output which labels the cell body, branch points and end points of the dendrites. The trace provides a skeletonization, or centerline, of the dendrite which we used to compute the linear network distances in the following analyses.

### Log-linear model as a tool for exploring important features and their dependencies

To find the most influential attributes with regard to prediction and spatio-temporal modeling of spines we fit a log-linear model to the feature data, which is a type of generalized linear model [26]. The co-occurrence frequencies of the features in question are essentially a large multidimensional contingency table of counts. The standard linear models assume that data is normally distributed around a certain mean, which means that the observations can take any real value, positive, negative, integer or fractional. Log-linear models, on the other hand, assume that data is intrinsically non-negative, typically counts that could be Poisson distributed, and allow us to model the association and interaction patterns among categorical variables. The attributes under consideration are BO, Type, SD and DIV. Again, since the type of spine was quite directly dependent on the length and the head diameter of the spine, we left these latter variables out of the modeling.

In order to analyze the data using a log-linear model, the various features must be in a categorical form or discretized. In an exploratory analysis such as this, one does not know what dependencies among features to expect; however we would like to note that these dependencies were not lost in the discretization process since trends in increasing and decreasing feature values would be preserved. To ensure that there were a reasonable number of observations at the higher branch orders, we pooled BO values of 5 or higher into a single category called "higher-order branches". We created a categorical variable to represent the continuous variable soma distance (SD) where categories were determined using the 4 quartiles of the SD spine data pooled over all 3 experiments. Specifically, SD values of less than 65.65 *μm* were classified into the first group, from this value to less than 108.99 *μm* the second, from this to less than 157.04 *μm* the third, and the rest (less than the most distal spine which lay at 413.25 *μm* from the cell body) fell in the fourth group. Binning the observed data for the continuous variables is the best way to get a general feel for how these quantities relate to each other. After this post-processing of the data we arrived at 5 categories of branch order, 4 categories of soma distance, 3 spine types (mushroom, stubby, and thin), and 3 DIVs (7, 14, and 21 days).

Using the observed frequencies for the aforementioned attributes, we created a four-way contingency table and fit the model using the 'glm’ function in the R package 'stats’. The table of the frequency of occurrences of the four attributes was modeled as Poisson with each entry being a simple count of the co-occurrences of that bin. We called this count *f*_*ijkl*_ with each of the subscripts *i*,*j*,*k*,*l* corresponding to a different attribute. The method uses the link function *y*_*ijkl*_ = *log*(*f*_*ijkl*_), and treats the model as a regular linear model. Each entry *y*_*ijkl*_ is modeled by a combination of coefficients: the intercept, plus main effects, plus every combination of interactions between these four attributes, as shown below.

(1) $$\begin{array}{ll} \;y_{\text{ijkl}} & =\mu +\alpha_{i}+\beta_{j}+\gamma_{k}+\delta_{l}+{\left(\alpha \beta \right)}_{\text{ij}}+{\left(\alpha \gamma \right)}_{\text{ik}}\\ & \;+{\left(\alpha \delta \right)}_{\text{il}}+\;\ldots \;+\;\text{error}. \end{array}$$

We estimated this full interaction model using the least-squares maximum-likelihood approach. We also used a stepwise fit algorithm, which begins with a model that includes only the constant term, and at each step chooses whether or not to add one additional term. The algorithm begins with the main effects then tries each possible 2-way interaction, aiming to minimize the Akaike Information Criterion (AIC). The AIC is defined as

(2) AIC=2k-2ln(L(θ|y,x))

where *k* is the number of parameters i.e the total number of coefficients being estimated, and

(3) $$L(\theta |y,x)=\underset{\theta }{\text{max}}\;\overset{N}{\underset{n=1}{\prod }}\frac{e^{y_{n}\theta^{\prime }x_{n}}e^{-e^{\theta^{\prime }x_{n}}}}{y_{n}!}$$

is the maximized value of the likelihood function for the estimated Poisson model. In the above equations $x=x_{1},\ldots ,x_{N}\in R^{4}$ are the input vectors, ***θ*** = *θ*_1_…*θ*_*k*_ are the parameter values (one per term in eqn. 1), and $y=y_{1},\ldots ,y_{N}\in R$ is the output. The AIC is a commonly used goodness-of-fit measure for a model given the observed data. Adding or subtracting terms, whether they be main effects, pairwise interactions, or up to 3-way interactions between attributes, will change the AIC value for the model. A lower AIC criterion indicates a better fit to the data and therefore a better model. To compute the stepwise fit we used the R function 'step’. For more information on the stepwise fit algorithm as well as the AIC criterion we ask that the readers refer to the 'step’ function reference ( [27], Chapter 6). We ran both of these LLM fitting procedures for all 3 experiments separately expecting to find general agreement between coefficients of the corresponding models created.

### Multinomial logistic regression to predict spine type from neighbor types

In order to predict spine type we first determined which attributes contributed most to spine type prediction. Given the complexity of the multidimensional LLM and the various interactions and conditional frequencies that would impinge on this issue, we decided to determine these attributes by analyzing 2-way contingency tables for spine type vs. SD, BO, DIV, as well as the spine types of the 3 nearest neighbors. This analysis helped us pick attributes that would be useful as the predictors in the multinomial logistic regression (MLR) [28] explained below.

When the response variable of a regression takes binary values "Logistic Regression" is used. This is an approach which uses a linear combination of the predictor variables to predict the log-odds of a success (the "logit" of the probability). Since our response variable was spine type and it can take 3 values (mushroom, stubby or thin), we needed to use a "Multinomial Logistic Regression" (MLR) which attempts to model the probability of any of multiple possible outcomes. We did not use the attributes SD or BO as predictors variables since the results of both the LLM analysis and 2-way contingency tables mentioned above told us that these quantities were not as relevant for spine type prediction. Therefore our model consisted of spine type as the output variable and the DIV, 1st, 2nd and 3rd nearest neighbor type along the dendrite as the predictor variables. We tried using only 1 or 2 nearest neighbors, however the results proved inconclusive because the prediction probabilities for each of the 3 types were predominantly close to 1/3. If we used more than the 3 nearest neighbors we sometimes ended up spanning a segment of dendrite which we did not consider to be "local", so we decided that 3 nearest neighbors provide the most useful information in the case of this study.

**Figure 2:**
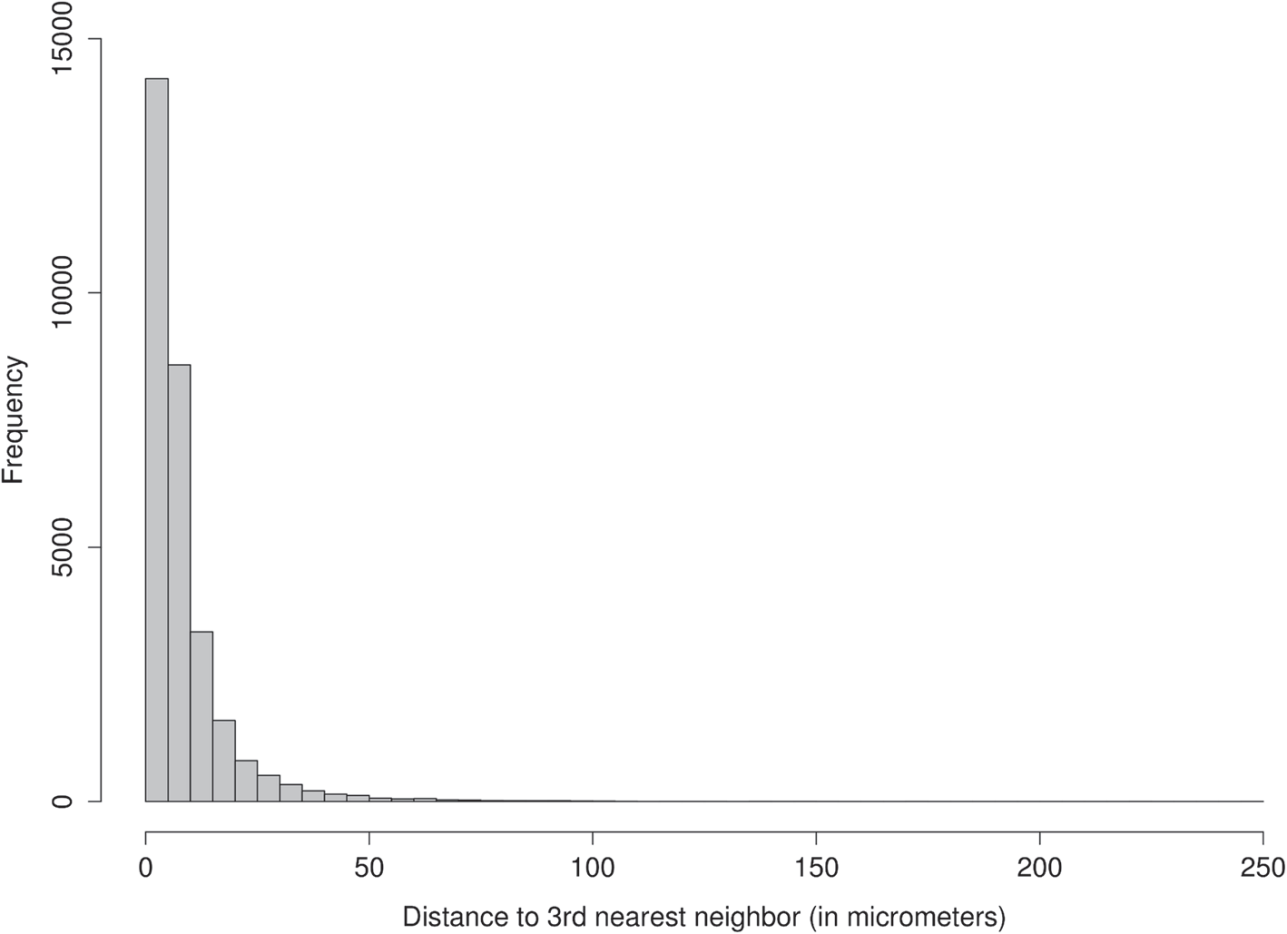
**Histogram of 3rd nearest neighbor distances.** This figure shows the distribution of 3rd nearest neighbor distances in order to get an idea of the physical neighborhood of spine types used for the MLR. It shows that although the maximum distance to any 3rd nearest neighbor was extremely high (248.31 *μm*) this distance was clearly an outlier case.

The MLR analysis we performed in this paper does disregard the actual inter-spine distances, meaning that if the 3 nearest neighbors are very close or very far apart we still treat them the same. We did this partially because adding the distance variables would complicate the model significantly, but also because we believe that over a large population of spines such as the one we have, these differences in distance will average out and we will still get a general picture of the trends between neighboring spine types. To verify that this was true we computed a histogram showing the distribution of 3rd nearest neighbor distances for each spine, shown in Figure 2. Although the maximum distance to any 3rd nearest neighbor is extremely high (248.31 *μm*) we can see from the histogram as well as the fact that the median 3rd nearest neighbor distance was 5.34 *μm* that this distance is clearly an outlier case and that the majority of 3rd nearest neighbor distances lie below 25 *μm*.

Suppose the output variable categories are denoted by 0,1,2 corresponding to mushroom, stubby or thin spines, with 0 being the reference category. If *y*_*i*_ denotes the observed outcome of the output variable (spine type), and *X*_*i*_ is the corresponding vector of the 3 neighbor types and DIV for the *i*th observation, one regression is run for the logit probability of each category *k*, with *β*_*k*_ representing the vector of regression coefficients in the *k*th regression (eqns. 4,5). This is done for all but the reference category, whose probability is then obtained by subtracting all other probabilities from one (eqn. 6). Note that because the predictor variables were spine types, which were nominal as opposed to ordinal variables, the predictor variables *X*_*i*_ must be represented with a "dummy coding". This means each neighbor type was represented by 2 predictor variables, where (1,0) corresponded to mushroom type, (0,1) corresponded to stubby type and (0,0) corresponded to thin type. This does not need to be done for the output variable *y*. With the addition of the DIV, which does not have to be dummy coded since it is an ordinal variable, this made each *X*_*i*_ vector of length 7.

The regressions are then written as:

(4) $$P(y_{i}=1)=\frac{\exp(\beta_{1}X_{i})}{1+\exp(\beta_{1}X_{i})+\exp(\beta_{2}X_{i})}$$

(5) $$P(y_{i}=2)=\frac{\exp(\beta_{2}X_{i})}{1+\exp(\beta_{1}X_{i})+\exp(\beta_{2}X_{i})}$$

and

(6) $$\begin{array}{ll} \;P(y_{i}=0) & =1-P(y_{i}=1)-P(y_{i}=2)\\ & =\frac{1}{1+\exp(\beta_{1}X_{i})+\exp(\beta_{2}X_{i})} \end{array}$$

The parameters are estimated typically by using an iterative procedure such as "iteratively re-weighted least squares" (IRLS) or, more commonly by a numerical approach (a quasi-Newton method) such as the Broyden-Fletcher-Goldfarb-Shanno (BFGS) method. In our case we create an MLR using the command *multinom* in the R package nnet [29] which uses BFGS by calling the R function *optim*. It can be seen that

(7) $$\text{log}(\frac{P(y_{i}=1)}{P(y_{i}=0)})=\beta_{1}X_{i}$$

(8) $$\text{log}(\frac{P(y_{i}=2)}{P(y_{i}=0)})=\beta_{2}X_{i}$$

so that the beta coefficients represent the change in the log odds of the dependent variable being in a particular category with respect to the reference category i.e. the thin type, for a unit change of the corresponding independent variable. To check if the models created from all three experiments were in agreement, we ran the MLR separately for each experiment.

To satisfy one of the major assumptions of this analysis, namely that the data must be a set of independent observations, we took 200 randomly sampled spines of each type from each experiment (600 spines per experiment total) to use for the parameter estimation. We chose to select equal proportions of each spine type in order to remove any bias in the model towards the less frequent thin spines, and 200 was the largest number we could justify using since there were only 649 thin spines in experiment 3. We verified that these randomly sampled spines did not lie within 10 *μm* of the image border so that we were fairly certain their nearest neighbors did not fall outside of the image plane. Note that due to the tortuosity of the dendritic structure this did not mean that our sample was necessarily biased towards spines which were proximal to the soma. We did not verify explicitly that the sampled spines were not neighbors of each other, since we assumed that the variation captured by the random sampling was enough to ensure some level of independence. The idea was to aim for an independent set of observations which represented the entire "population" of spines in that experiment. To be clear we used all 30,285 spines for the LLM model and K-function analysis, only the MLR model required random sampling since we were using neighbor information which would have been redundant if we considered every spine.

To verify that the prediction of spine type provided by the MLR was better than what we would get purely by their relative abundance i.e. without neighboring spine type information, we computed something similar to a "Bayes Factor" [30]. Bayes factor is a method of choosing between two models on the basis of the observed data. In our case, the first prediction model was simply the prior global probability of finding a given spine type based on its frequency in the particular experiment under consideration. The second model was the MLR prediction model using the neighbor type information. We computed *P*(*Y* = *i*|*X*)/*P*(*Y* = *i*) and reasoned that values considerably larger than one indicated the neighboring spine type information was helpful in the prediction of the central spine type.

### Linear network K-function as a tool for testing spatial point patterns

Originally proposed by Ripley in 1981 [31], the purpose of the K-function is to estimate whether or not there is clustering or repulsion present in a given spatial point process. The common null hypothesis is that the points within the observation window are distributed as a homogeneous Poisson process, which is also termed "completely spatially random" or CSR. This means that the density of points does not vary depending on the spatial parameters i.e. x and y in the 2D Euclidean case, or the location along the dendritic network in our case. In order to determine if this is a valid null hypothesis for our data, we created Q-Q plots [32] for individual dendrites which compared the quantiles of the SD values of observed spines to the theoretical quantiles for the CSR case. If the two distributions (observed and CSR) being compared were similar, the points in the Q-Q plot would approximately lie on the line *y* = *x*. In order to create the theoretical quantiles it is necessary to know the values of SD at any location on the given network, not just at the spine locations. Once we have this we can partition the network into epsilon small segments and assign each segment a value 1 if it contains a spine and 0 otherwise based on the CSR assumptions. We did this using code provided to us by Adrian Baddeley and Gopal Nair at the Commonwealth Scientific and Industrial Research Organization (CSIRO), Australia.

The K-function computes the expected number of points within a distance *t* of an arbitrary point *p*, therefore the empirical value in 2D Euclidean space for the CSR case will be proportional to the circular area, *λπt*^2^. The proportionality constant *λ* represents the density of points in the homogeneous Poisson case, and can be estimated by finding the total number of points *N* divided by the total area of the observation window *A*. Ripley’s K-function, which is a function of *t*, is a very useful tool because it describes the 2^*nd*^ order characteristics of the point process at several scales *t*. If we ignore the edge effects due to the observation window, the observed $\hat{K}(t)$ can be written as:

(9) $$\hat{K}(t)=\frac{\left|A\right|}{N^{2}}\underset{i}{\sum }\;\underset{j\neq i}{\sum }I(d_{\text{ij}}<t)$$

where *I* stands for the indicator function, and *d*_*ij*_ stands for the Euclidean distance between two points *p*_*i*_ and *p*_*j*_. In the above equation, we see that the expectation is normalized by 1/*λ* since $\lambda =\frac{N}{\left|A\right|}$, so we infer that theoretically *K*(*t*) = *πt*^2^ implies spatial independence of points, or a CSR point process. Therefore, if *K*(*t*) is the theoretical CSR value of the function and $\hat{K}(t)$ is the observed function, then $\hat{K}(t)>K(t)$ implies clustering between points and $\hat{K}(t)<K(t)$ implies repulsion. It is possible to extend this function to multi-type point patterns (i.e. to find clustering or repulsion between specific spine types) or to higher dimensional data (i.e. space-time, or 3D Euclidean space).

**Figure 3:**
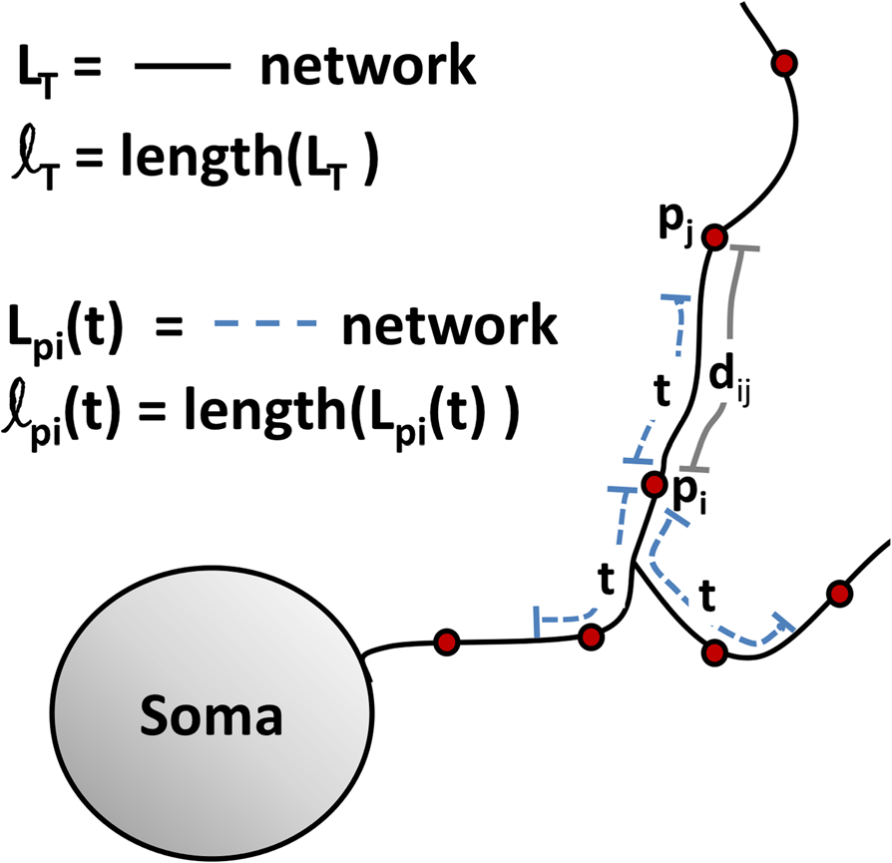
**Visualization of the linear network K-function.** This figure clarifies what is meant by the quantities *d*_*ij*_, *L*_*T*_, *ℓ*_*T*_, $L_{p_{i}}(t)$, and $\ell_{p_{i}}(t)$ which were used to compute the linear network K-function. Here *d*_*ij*_ is the linear network distance shown by the gray line between points *p*_*i*_ and *p*_*j*_. *L*_*T*_ (in black) is the entirety of the single dendritic network and *ℓ*_*T*_ is the length of *L*_*T*_. Similarly, $L_{p_{i}}(t)$ (in blue dashed lines) is subset of the network where the distance between a point *p*_*i*_ and any other point is ≤ *t* and $\ell_{p_{i}}(t)$ is the length of $L_{p_{i}}(t)$. In this particular example there are 2 spines which fall within $L_{p_{i}}(t)$ and would be counted in determining the empirical function value $\hat{K}(t)$, however point *p*_*j*_ falls outside this radius and would therefore not be counted.

Since our particular point process consists of spines which lie along the "linear network" of the dendritic tree we were primarily concerned with inter-spine distances along the dendrite as opposed to in Euclidean space. Therefore we used a version of the K-function developed recently for linear networks by Okabe and Yamada [15]. This modified version of the K-function takes into account the structure of the linear network on which the point process resides and imitates the Euclidean space K-function described above. The linear network K-function is calculated as follows:

(10) $$\hat{K}(t)=\frac{\ell_{T}}{N^{2}}\overset{N}{\underset{i=1}{\sum }}\underset{j\neq i}{\sum }I(d_{\text{ij}}<t)$$

where *ℓ*_*T*_ is the length of the total network *L*_*T*_. The theoretical CSR for this case is described as follows:

(11) $$K(t)=\frac{1}{\ell_{T}}\int_{p\in L_{T}}\ell_{p}(t)\text{dp}$$

where *p* is a point belonging to the set of all points *P* = {*p*_1_,…,*p*_*N*_}, and *ℓ*_*p*_(*t*) is the length of the subset of the network *L*_*p*_(*t*) where the distance between p and any other point is ≤ *t*. Note that here the distance *d*_*ij*_ stands for the linear network distance along the dendrite. Accounting for variability in the length *ℓ*_*p*_(*t*) means the formula takes into account the edge effects due to the observation window (in our case the image plane) inherently, but at the cost of added complexity. The computation of the theoretical linear network K-function requires us to find $L_{p_{i}}(t)$, the subset of *L*_*T*_ where the network distance between a specific point *p*_*i*_ and any other point is ≤ *t*, and $\ell_{p_{i}}(t)$, the length of that subset, for every point *p*_*i*_. A visualization of the quantities *d*_*ij*_, *L*_*T*_, *ℓ*_*T*_, $L_{p_{i}}(t)$, and $\ell_{p_{i}}(t)$ is shown in Figure 3.

Note that although many biological applications of point processes treat individual observations as replicate patterns coming from the same underlying distribution, we cannot do that using the above definition of the network linear K-function due to the change in linear network structure from dendrite to dendrite. The term "dendrite" here refers to the entire dendritic tree resulting from a single root branch of a neuron. Other in-vivo studies [33,34] focus on clustering of spines which lie on the same unbranched section of the dendrite, however we focus on the entire dendritic tree under the hypothesis that it follows rule-based distributions of spines due to anatomical constraints and integration of the a signal over the entire dendrite. One can infer from Figure 3 that since the geometry of the linear network changes from dendrite to dendrite, so do the total lengths of the networks *ℓ*_*T*_, the ranges of possible t-values and the amount of dendritic length that is present within a given distance of any point. We did not simply normalize the lengths of the networks to a [ 0,1] scale because it is desirable for the t-axis to retain its real physical values in order to make conclusions about the scale (in *μm*) of clustering or repulsion among spines. However, we did desire to compare the linear network K-functions of various dendrites in a meaningful way. For this reason we used a corrected version of the network K-function that intrinsically compensates for the geometry of the network called Ang’s correction [35]. The observed K-function then becomes:

(12) $$\hat{K}(t)=\frac{\ell_{T}}{N(N-1)}\overset{N}{\underset{i=1}{\sum }}\underset{j\neq i}{\sum }\frac{I(d_{\text{ij}}\leq t)}{m(i,d_{\text{ij}})}$$

where *m*(*i*,*d*_*ij*_) is the number of points of *L* lying at the exact distance *t* away from the point *i* measured by the shortest path. That is, the contribution to the function from each pair of points (*i*,*j*) is weighted by the reciprocal of the number of points that are situated at the same distance from *i* as *j* is. As a result, the theoretical CSR case is simply *K*(*t*) = *t* for all 0 ≤ *t* < *T*. This enables direct comparison of t-values across dendrites, as we will see in the results section.

#### Simulations and q-values

To test the null hypothesis that the locations of spines on the dendrites were indeed CSR, we created a summary statistic which encompasses the difference between the empirical $\hat{K}(t)$ and the theoretical *K*(*t*) under CSR. The summary or "test statistic" we used, is the max absolute difference (MAD) over t, viz.

$$d=\underset{t}{\text{max}}|K(t)-\hat{K}(t)|.$$

One method for obtaining a distribution of *d* proposed by Diggle [36] is to bootstrap the residuals, or differences between the observed and theoretical values. However a more heuristic and intuitive way is to simulate the CSR case for each dendrite, compute the K-function for each of these simulations, and find the simulated distribution of our test statistic. We then found the p-value of the observed difference *d* from this simulated distribution.

Specifically, we carried out 1000 CSR simulations for each dendrite by placing uniform points on a line [0,*ℓ*_*T*_], and mapping them to that specific dendrite’s linear network structure. The number of points simulated per dendrite equaled the number of observed spines for that dendrite, thus preserving the overall density *λ*. This means the same number of spines that existed on each dendrite were randomly placed along the linear network specific to that dendrite. We used these simulations to obtain 1000 values of the summary statistic, say *d*[*i*]. Then the p-value for each dendrite was simply the proportion of simulated values that fell above the observed or experimental value of *d*, i.e. the rank of this *d* within the 1000 values of *d*[*i*], or *nrank*/(*nsim* + 1).

This p-value approach is similar to the test which rejects the null hypothesis if the graph of the observed K-function lies outside the "point-wise simulation envelope" at any value of t. A simulation envelope is essentially a graphical measure of how far a function can deviate from the theoretical value without being considered significant at a given level. As mentioned above in our case the envelope is calculated by first creating the 1000 CSR simulations of a point pattern on a given dendritic network with the same observed network intensity, then calculating the linear K-function for each of these 1000 simulations. To perform a two-sided significance test at the 10% level, the 5% and 95% percentiles are then calculated based off the 50 lowest and 50 highest linear K-function values *per t-value*, hence the term "point-wise". Plotting these values as a function of t gives one a visual idea of the spread that is produced by chance mechanisms alone. If the observed K-function for a given t-value does not fall outside these percentiles, it is considered insignificant for that t-value at the 10% significance level. We make use of the R package 'spatstat’ [18] for obtaining the point-wise simulation envelope.

Because we have a multitude of hypothesis tests and p-values (one for each dendrite), to reach a conclusion about the general trend for each DIV and experiment, we used the concept of False Discovery Rate (FDR) [37]. The FDR is defined as

(13) $$\pi_{0}=\frac{\#\;\text{true}\;\text{null}\;\text{tests}}{\#\;\text{total}\;\text{tests}}$$

Controlling the overall FDR, or expected proportion of incorrectly rejected null hypotheses termed "false discoveries", is a statistical method commonly used in multiple hypothesis testing which increases the statistical power of each test. What is more general and useful however, is a test-specific FDR measure. This essentially allows us to look at all possible significance thresholds at once, as well as provide each test with a measure of significance that can be easily interpreted. This is accomplished by calculating an analogue of the p-value for each test called a "q-value" [38]. A p-value of 0.05 implies that 5% of all tests will result in false positives, whereas a q-value of 0.05 implies that 5% of *significant* tests will result in false positives. Since the latter is clearly a far smaller quantity, q-values generally indicate fewer significant tests than p-values for a given significance threshold and provide a far more accurate indication of the level of false positives in the case of multiple hypothesis testing. For q-value estimation we used the qvalue package available from [39].

## Results and discussion

### Data analyzed

We performed three biological replicate experiments resulting in a total of 75 neurons from the following time points: DIV 7, DIV 14, and DIV 21 (Table 1). This provided a rich and complete data set resulting in 485 dendritic branches and 30,285 spines. Example images from each DIV along with zoomed in dendritic segments where spines and annotations are visible are shown in Figure 1. Scale bars are shown in red in panels A-C and the yellow rectangular boxes in panels A-C show the region of interest which has been zoomed in on in panels D-F respectively. Panels D-F are all at the same resolution.

**Table 1:** Number of neurons collected per experiment and DIV

**EXP**	**DIV7**	**DIV14**	**DIV21**
1	8	9	7
2	10	10	10
3	7	7	7

**Figure 4:**
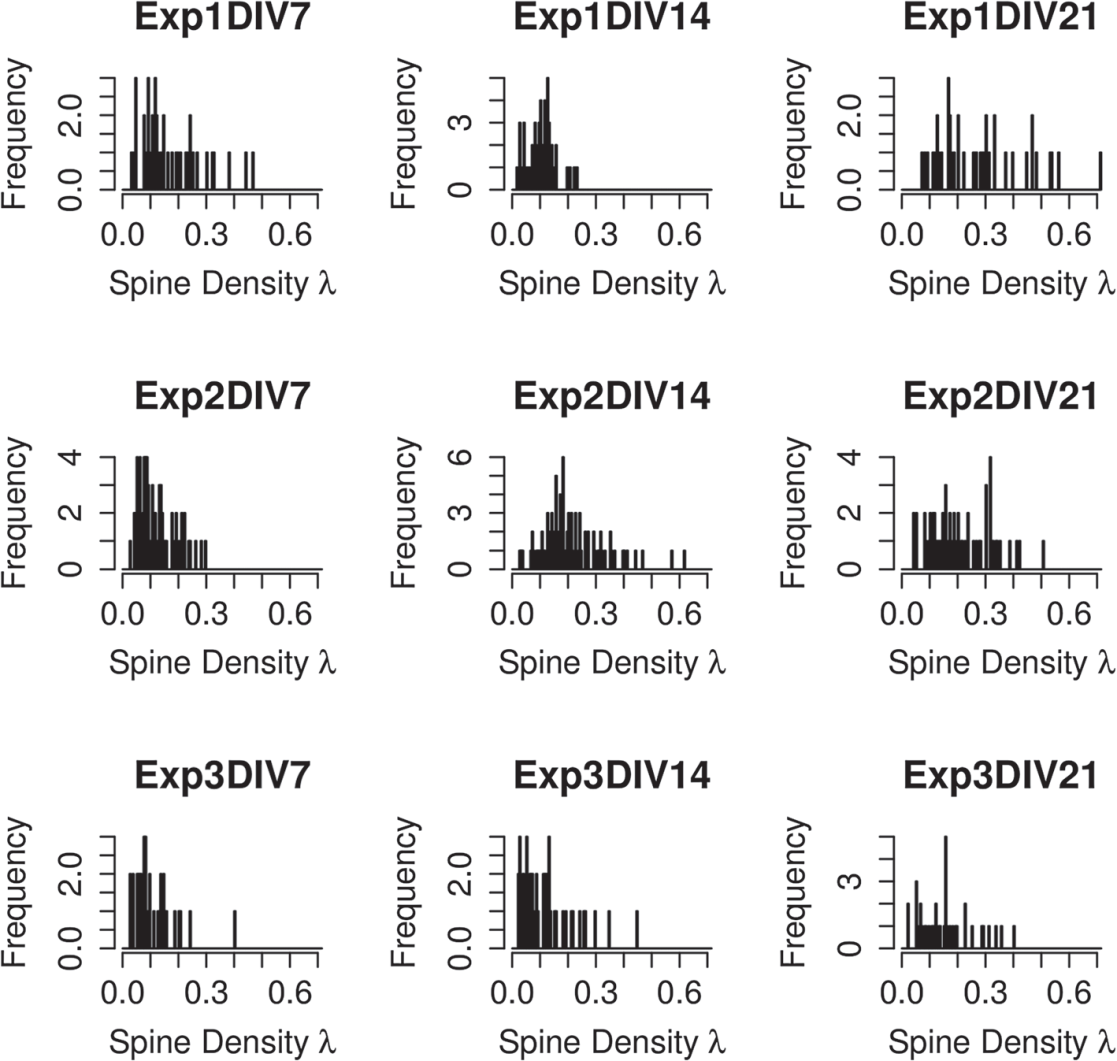
**Histograms of spine density per dendrite for each experiment and DIV.** This figure shows histograms of the number of spines per *μm*, or *λ*, for each dendrite in different experiments and time points.

The number of spines per *μm*, or *λ*, for each dendrite in different experiments and time points is shown in Figure 4. We chose to include this in order to help the reader compare these neuronal culture results with other experimental paradigms with which they may be more familiar. It is clear from the histograms that the distribution of spine density for DIV7 is skewed toward lower values as compared to the density for DIV21, as expected. The image data as well as spine and trace annotations are made publicly available through the BISQUE system [40] and the URL is given in the section titled "Availability of supporting data". We chose BISQUE over other databases like NeuroMorpho.Org [41] because it allows us to upload multiple layers of annotations as opposed to only the digital reconstruction files.

We calculated a 2-way contingency table over all experiments and spine types and obtained Table 2. From this table we note the high frequency of mushroom and stubby spines as compared to thin spines, and also the fact that the ratio of types does not remain the same per experiment even though they were indeed biological replicates. In fact, a Pearson’s Chi-Squared test on Table 2 shows dependence between the spine type counts and experiment number, *χ*^2^(*df* = 4,*N* = 30285) = 659.87, *p* < 0.0001.

**Table 2:** Number of each type of spine per experiment

**EXP**	**Mushroom**	**Stubby**	**Thin**
1	4035	3224	1915
2	5400	6619	2570
3	2388	3485	649

We believe that the large experimental variation between spine type proportions and counts in each experiment was a positive result because this meant that statistical agreement across all 3 experiments relating to spine type clustering and density estimation carries heavier weight than if the 3 experiments were more uniform in these quantities, or if we had pooled data from all 3 experiments together. Also, if all 3 experiments were unusually homogeneous there could be a possibility that it is a result of our specific culturing, imaging or spine extraction methods rather than a true representation of the underlying biological process. The various biological systems to which these techniques will be applied will certainly have this type of variability.

### Spine type is independent of distance from soma

As described in the Methods section, we calculated a stepwise-fit of the log-linear model starting with just a constant term, and at each step choosing to add the main effects (div, type, bo and sd) and possible 2-way interactions between main effects one-by-one if they decreased the corresponding AIC value. The captions above Tables 3, 4 and 5 show the final models arrived at for each of the 3 experiments as well as their corresponding AIC values. The tables indicate the change in the AIC value that would occur from adding or omitting each of the terms in the first column. This gives us an idea of how important that term was to the model. The rows of the table are ordered by their overall contribution to the model, i.e. the term in the first column of the first row of each table had the lowest AIC value and was therefore the most important to the overall model. If the reader requires further information on the AIC criterion or how to interpret this table we ask them to refer to Chapter 6 of [27].

**Table 3:** EXP 1 stepwise final model: freq ∼ div + type + bo + sd + bo ·sd + div ·bo + div ·type + div ·sd + type ·bo + type ·sd, AIC = 1557.05

	**Df**	**Deviance**	**AIC**
None		530.4	1557.1
Omit type ·sd term	6	545.0	1559.6
Omit type ·bo term	8	558.8	1569.4
Omit div ·sd term	6	569.6	1584.2
Omit div ·type term	4	648.0	1666.6
Omit div ·bo term	8	1324.1	2334.7
Omit bo ·sd term	12	4142.4	5145.0

**Table 4:** EXP 2 stepwise final model: freq ∼ div + type + bo + sd + bo ·sd + div ·bo + div ·sd + div ·type, AIC = 1243.13

	**Df**	**Deviance**	**AIC**
None		470.2	1243.1
Add type ·sd term	6	461.3	1246.3
Add type ·bo term	8	465.5	1254.4
Omit div ·type term	4	610.4	1375.3
Omit div ·sd term	6	696.0	1456.9
Omit div ·bo term	8	906.5	1663.5
Omit bo ·sd term	12	5208.2	5957.1

**Table 5:** EXP 3 stepwise final model: freq ∼ div + type + bo + sd + bo ·sd + div ·sd + div ·type + div ·bo + type ·sd + type ·bo, AIC = 1441.29

	**Df**	**Deviance**	**AIC**
None		482.24	1441.3
Omit type ·bo term	8	522.95	1466.0
Omit type ·sd term	6	542.08	1489.1
Omit div ·bo term	8	606.34	1549.4
Omit div ·type term	4	630.62	1581.7
Omit div ·sd term	6	715.38	1662.4
Omit bo ·sd term	12	2825.69	3760.7

Despite the fact that they were included in the final stepwise fit model for experiments 1 and 3, the AIC values in Tables 3, 4 and 5 show that in all 3 experiments the interaction between spine type and soma distance ("type ·sd") as well as spine type and branch order ("type ·bo") were the least important in modeling the overall frequency table of occurrences. This implies that the correlation between these quantities was not very high, therefore we reason that it was not necessary to use either SD or BO to predict the spine type in the MLR created in the following section. We also noticed that the term marking the interaction between BO and SD was the most important pairwise term in all stepwise fit models. It is expected that BO and SD are correlated because both necessarily increase as we move away from the cell body. Indeed, running a 2-way Chi-square test on the contingency table of the discretized versions of these variables showed us high dependence, *χ*^2^(*df* = 12, *N* = 30285) = 11635.19,*p*<0.0001. We also saw a high level of dependence between DIV and SD (*χ*^2^(*df* = 6, *N* = 30285) = 681.76, *p* < 0.0001) and between DIV and BO (*χ*^2^(*df* = 8, *N* = 30285) = 1604.75, *p* < 0.0001). This was intuitive as well since we expect both BO and SD to generally increase with DIV.

It is possible that the Type vs. SD relationship could have also been estimated using a Sholl-type analysis ( [42]) where we count the number of each type occurring within concentric circles from the soma and verify that it is constant, however this would not necessarily produce the same results. The crucial difference between our approach and the Sholl approach is that in our approach the "distance from soma measures" the actual distance along the centerline of the dendrite instead of the radial distance from the cell center. This is especially important for dendrites with high tortuosity (which we find prevalent in our data), since the radial distance in those cases will not correspond to the dendritic distance from the cell body. Many studies of cultured neurons use Sholl analysis, however they use it in its original form for counting dendritic intersections and do not comment on the relation to spine density or type. To our knowledge this is the first study to quantify the spine density vs. distance to the soma in dissociated neuronal cultures.

Three-way and 4-way interactions are generally known to be weak (not as explanatory as the main effects and 2nd order interactions) and difficult to interpret, however in the interest of exploring all possibilities we computed the maximum likelihood fit using all 4 attributes as well as a stepwise fit model which allows for 3-way interactions between attributes. The table presented in Additional file 1 results from the LLM which models all possible interactions of all 4 attributes, i.e. up to the fourth order. The coefficients presented in the table are those which were significant at the 0.1*%* level, and the corresponding p-values are shown in the last column. The table contains the interactions which were more important to the model, and shows that of these interactions only one (highlighted in green) between type and either BO or SD, was shown as being significant over all experiments. This verifies once again that neither BO nor SD were highly correlated with the spine type. In addition to this, the stepwise fit models in Additional file 2 show that if we did allow 3rd order interactions, the strongest 3rd order correlation over all experiments was that of DIV, SD and BO, again affirming that all 3 of these quantities should intuitively increase together.

### Spines tend to cluster with other spines of the same type

In creating a regression model, we first ascertain that the predictor variables used are not only useful in predicting the output variable, but also that they do not provide redundant information as this can throw off the model fitting process. Using all spines in the dataset, we performed a Chi-square test on the 2-way contingency tables of spine type versus binned SD and BO, DIV, and the types of the 3 nearest neighbors (N1, N2, N3) as described in the Log-Linear Model section above. Due to the aforementioned dependence between the type and experiment number we performed the test separately for each experiment and the results are shown in Table 6. From the table we can see that the DIV and the 3 nearest neighbors showed clear dependency with spine type in all experiments, whereas SD and BO showed independence at the 5% significance level in experiments 1 and 2 respectively. Since we expected SD and BO to have a similar relationship with type due to the high correlation mentioned above, and we had found this was not a very strong relationship, we chose to use only DIV, N1, N2 and N3 as predictors for spine type in the MLR model.

**Table 6:** Chi-square results for spine type vs. other attributes

	**EXP1, *N * = 9174**	**EXP2, *N * = 14589**	**EXP3, *N * = 6522**
Type ·SD, *df* = 6	*χ*^2^ = 9.13,*p* = 0.1665	*χ*^2^ = 33.64,*p* < 0.0001	*χ*^2^ = 25.08,*p* = 0.0003302
Type ·BO, *df* = 8	*χ*^2^ = 29.02,*p* = 0.0003147	*χ*^2^ = 12.39,*p* = 0.1348	*χ*^2^ = 26.53,*p* = 0.0008516
Type ·DIV, *df* = 4	*χ*^2^ = 119.78,*p* < 0.0001	*χ*^2^ = 358.25,*p* < 0.0001	*χ*^2^ = 139.28,*p* < 0.0001
Type ·N1, *df* = 4	*χ*^2^ = 225.93,*p* < 0.0001	*χ*^2^ = 212.87,*p* < 0.0001	*χ*^2^ = 246.74,*p* < 0.0001
Type ·N2, *df* = 4	*χ*^2^ = 163.67,*p* < 0.0001	*χ*^2^ = 226.31,*p* < 0.0001	*χ*^2^ = 127.91,*p* < 0.0001
Type ·N3, *df* = 4	*χ*^2^ = 90.33,*p* < 0.0001	*χ*^2^ = 153.11,*p* < 0.0001	*χ*^2^ = 131.96,*p* < 0.0001

The resulting beta coefficients for each of the predictor variables are shown in Table 7. Here "N1-Var1" refers to the beta coefficent of the first dummy variable for the type of the first nearest neighbor; "N1-Var2" refers to the second dummy variable, and so on. The "mushroom" row is omitted because it is the reference category and its probability is obtained as shown in eqn. 6. We computed the prediction probabilities for each spine type given each combination of neighbor types for each experiment separately to determine the agreement between experiments. A selected set of results are shown below in Tables 8, 9 and 10. The highest probability for each row is marked by an asterisk. Note that in these tables all DIVs in all experiments predicted the spine type to be mushroom when its 3 nearest neighbors were mushroom type, and stubby when the 3 nearest neighbors were stubby type. Thin types were the most probable when the three nearest neighbors were thin type in all but experiment 2 DIV14 and DIV21. The probabilities for cases where all 3 of the nearest neighbors were not of the same type have been omitted for brevity and because they did not show any clear trends.

**Table 7:** MLR beta coefficients for all 3 experiments

**EXP1**	**(Intercept)**	**N1-Var1**	**N1-Var2**	**N2-Var1**	**N2-Var2**	**N3-Var1**	**N3-Var2**	**DIV**
Stubby	0.06	0.04	0.47	-0.52	0.10	0.09	0.25	-0.01
Thin	1.05	-0.57	-0.34	-0.84	-0.57	-0.23	-0.32	0.00
**EXP2**	**(Intercept)**	**N1-Var1**	**N1-Var2**	**N2-Var1**	**N2-Var2**	**N3-Var1**	**N3-Var2**	**DIV**
Stubby	0.08	0.03	0.67	-0.14	0.05	-0.20	-0.09	-0.02
Thin	0.25	-0.76	-0.17	-0.61	-0.37	-0.06	-0.05	-0.02
**EXP3**	**(Intercept)**	**N1-Var1**	**N1-Var2**	**N2-Var1**	**N2-Var2**	**N3-Var1**	**N3-Var2**	**DIV**
Stubby	-0.36	-0.24	0.33	-0.14	0.19	-0.03	0.30	0.01
Thin	0.35	-0.66	-0.58	-0.33	-0.28	-0.25	-0.33	-0.02

**Table 8:** Prediction Probabilities: N1 = mushroom, N2 = mushroom, N3 = mushroom

**DIV7**	**EXP**	**P(mushroom)**	**P(stubby)**	**P(thin)**
	1	0.45*	0.30	0.25
	2	0.51*	0.35	0.13
	3	0.54*	0.27	0.20
**DIV14**	**EXP**	**P(mushroom)**	**P(stubby)**	**P(thin)**
	1	0.45*	0.28	0.26
	2	0.55*	0.33	0.12
	3	0.54*	0.28	0.18
**DIV21**	**EXP**	**P(mushroom)**	**P(stubby)**	**P(thin)**
	1	0.46*	0.27	0.27
	2	0.59*	0.30	0.11
	3	0.54*	0.30	0.16

^*^ denotes the highest probability per row.

**Table 9:** Prediction probabilities: N1 = stubby, N2 = stubby, N3 = stubby

**DIV7**	**EXP**	**P(mushroom)**	**P(stubby)**	**P(thin)**
	1	0.24	0.55*	0.21
	2	0.30	0.52*	0.18
	3	0.32	0.55*	0.12
**DIV14**	**EXP**	**P(mushroom)**	**P(stubby)**	**P(thin)**
	1	0.25	0.53*	0.22
	2	0.33	0.50*	0.17
	3	0.32	0.58*	0.11
**DIV21**	**EXP**	**P(mushroom)**	**P(stubby)**	**P(thin)**
	1	0.26	0.51*	0.23
	2	0.37	0.47*	0.16
	3	0.31	0.60*	0.09

^*^denotes the highest probability per row.

**Table 10:** Prediction Probabilities: N1 = thin, N2 = thin, N3 = thin

**DIV7**	**EXP**	**P(mushroom)**	**P(stubby)**	**P(thin)**
	1	0.20	0.20	0.60*
	2	0.33	0.31	0.36*
	3	0.33	0.25	0.42*
**DIV14**	**EXP**	**P(mushroom)**	**P(stubby)**	**P(thin)**
	1	0.20	0.19	0.61*
	2	0.37*	0.30	0.34
	3	0.34	0.27	0.39*
**DIV21**	**EXP**	**P(mushroom)**	**P(stubby)**	**P(thin)**
	1	0.20	0.17	0.62*
	2	0.41*	0.28	0.32
	3	0.34	0.30	0.36*

^*^ denotes the highest probability per row.

The Bayes factor results in Table 11 show that the proportional gain in information for the spine type in question was always greater than one for the prediction of a particular type when the neighborhood types were all of that same type. Due to the low frequency of thin spines, their corresponding Bayes factors were higher than that of other types, meaning that their prediction probabilities benefit more than other types from neighborhood type information.

**Table 11:** Bayes factors

**BF(mushroom): N1 = mushroom, N2 = mushroom, N3 = mushroom**			
EXP	DIV7	DIV14	DIV21
1	1.02	1.03	1.05
2	1.39	1.49	1.60
3	1.47	1.47	1.47
**BF(stubby): N1 = stubby, N2 = stubby, N3 = stubby**			
EXP	DIV7	DIV14	DIV21
1	1.56	1.50	1.44
2	1.15	1.10	1.05
3	1.03	1.08	1.12
**BF(thin): N1 = thin, N2 = thin, N3 = thin**			
EXP	DIV7	DIV14	DIV21
1	2.85	2.91	2.98
2	2.04	1.92	1.79
3	4.22	3.87	3.54

### Dendritic spine densities are completely spatially random

**Figure 5:**
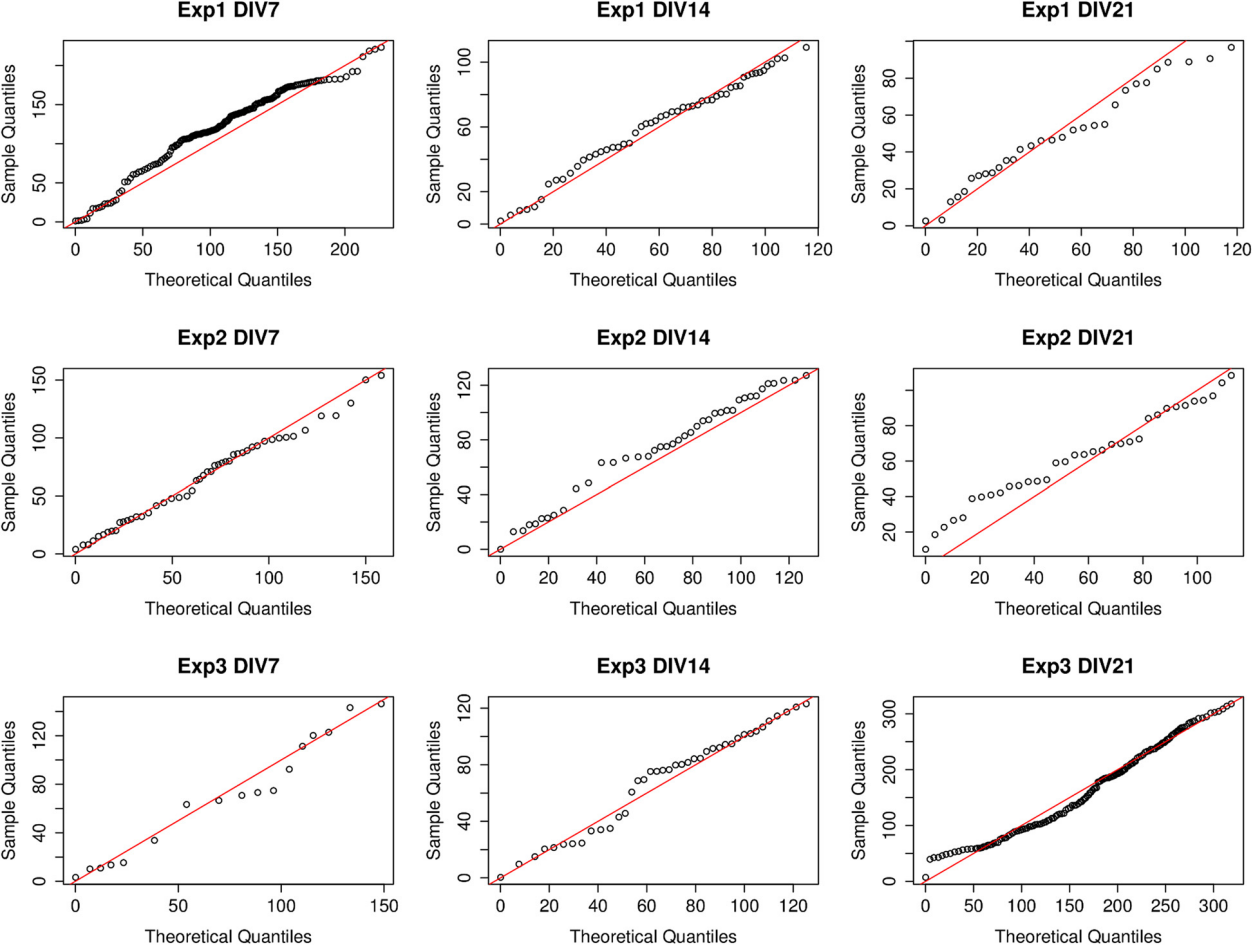
**Q-Q Plots of spine density vs. soma distance for a set of 9 example dendrites.** This figure presents the Q-Q plots of spine density vs. distance from soma for 9 (of the 485) example dendrites. We randomly selected 1 dendrite from each DIV and each biological replicate (experiment) to ensure the diversity of the set. The *y* = *x* line is marked in red, and the observed Q-Q values are marked as black circles. Visual inspection of these plots show that they follow the line *y* = *x* closely enough to assume that the spine locations being CSR was a viable null hypothesis.

We created Q-Q plots as described above based on the quantiles of spine counts vs. distance from the soma and found that upon visual inspection almost all dendrites follow the theoretical uniform distribution closely enough to assume that the density of the spines was homogeneous and therefore the CSR case was a viable null hypothesis. We selected 9 (out of 485) example dendrites and their Q-Q plots are shown in Figure 5. We randomly selected 1 dendrite from each DIV and each biological replicate (experiment) to ensure the diversity of the set. The *y* = *x* line is marked in red, and the observed Q-Q values are marked as black circles. Note that because this is a graphical method for comparing two probability distributions there was no p-value or significance level associated.

**Figure 6:**
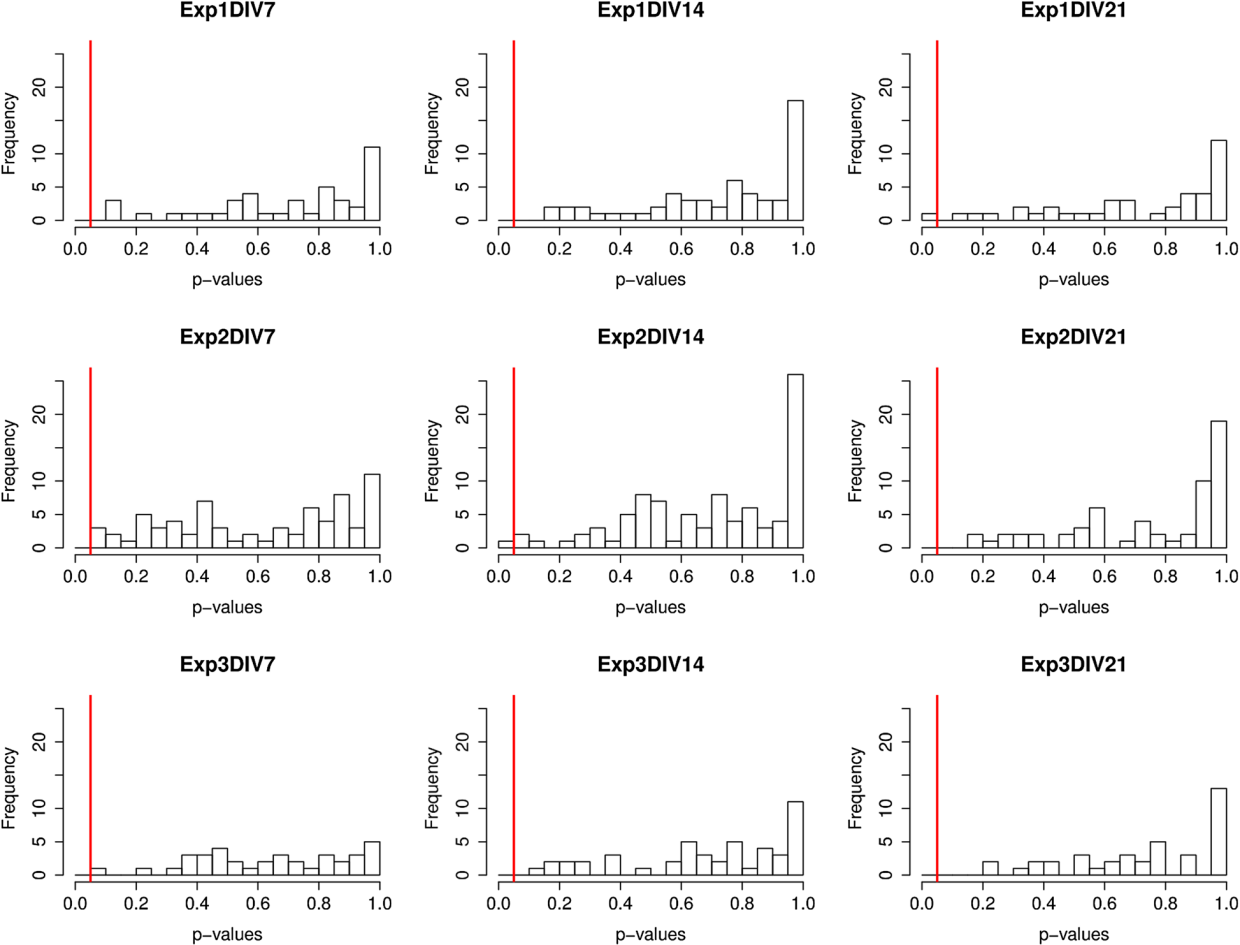
**P-values of linear network K-function MAD statistic for each experiment and DIV.** This figure shows histograms of all dendrite p-values per experiment and DIV before FDR was applied. In each case the 5% significance level is marked by a red vertical line. Q-values were not included as a separate figure because they were all zero.

Of all the 485 dendrites analyzed, only three of them (Exp. 1 DIV 21, Exp. 2 DIV 14, and Exp. 2 DIV 21) were considered non-CSR at the 5% significance level. Figure 6 shows histograms of the p-values of all 485 dendrites separated into each DIV and experiment number. The 5% significance level is shown by the red vertical line in each case. We then computed the q-values for each dendrite and found that they are all equal to 1. This is not surprising according to the explanation of the q-value above. Recall that q-values equal to 1 imply that 100% of the significant tests resulted in false positives, i.e. there were no significant tests. We therefore conclude that regardless of the maturity of the neuron, or the variation over biological replicate experiments, the locations of spines along all of the dendrites we analyzed were completely spatially random.

**Figure 7:**
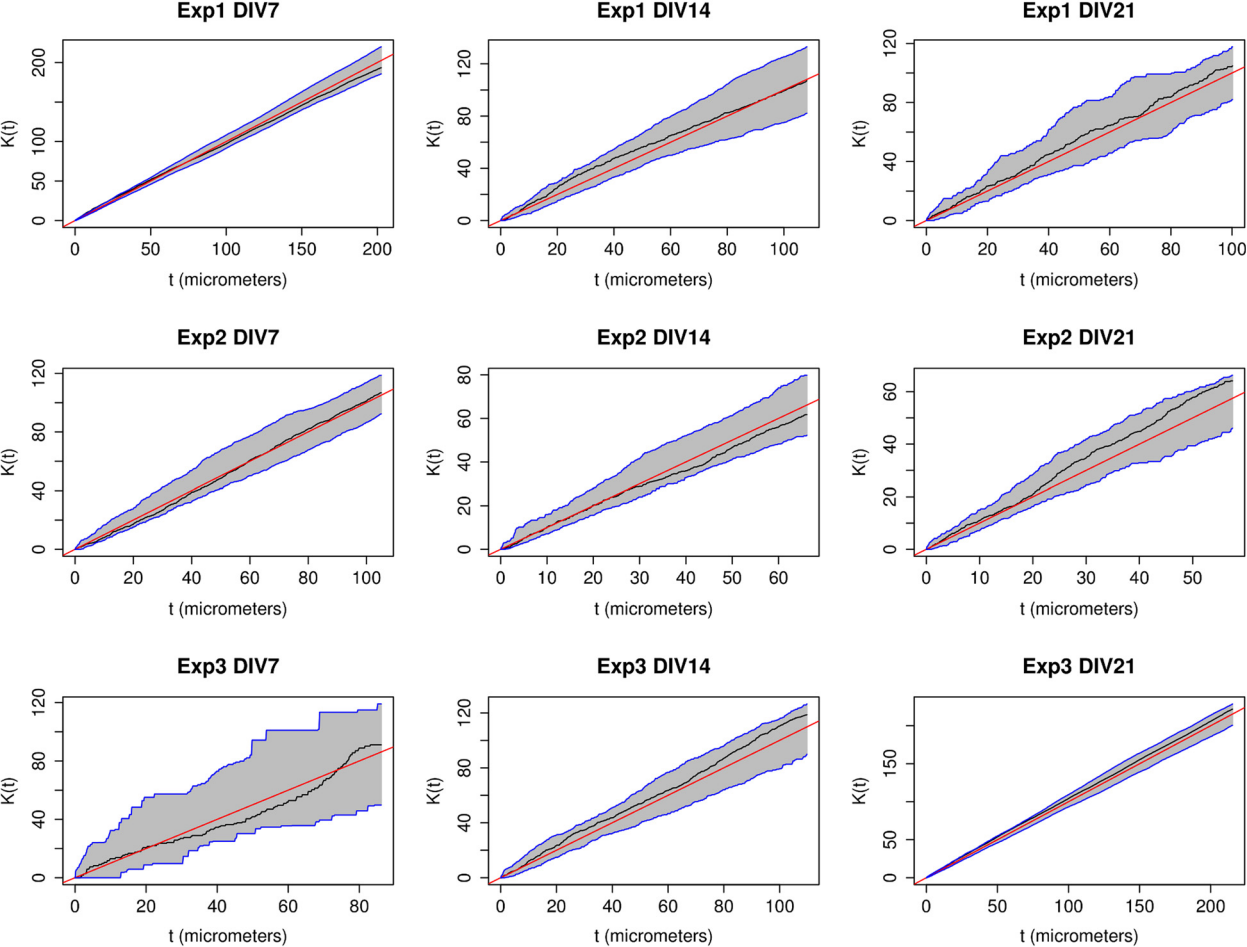
**Theoretical and observed K-functions and simulation envelopes for a set of 9 example dendrites.** This figure shows the K-function for the same 9 (of 485) example dendrites used for the QQ-plots of Figure 5. We randomly selected 1 dendrite from each DIV and each biological replicate (experiment) to ensure the diversity of the set. Each graph shows the observed $\hat{K}(t)$ function (black), the theoretical *K*(*t*) function (red) as well as the two-sided 5% and 95% point-wise simulation envelopes as a function of the radius *t*. We see here that the black curves do not leave the gray shaded area for any value of t, which means that the deviation from spatially random is insignificant at the 10% level for every t-value.

As mentioned above, the K-function is a function of the inter-point distance, *t*, that we consider around each observed point. The range of t-values is determined by the total length of the network *ℓ*_*T*_, therefore because each dendrite has a different network length it also has a different range of t-values. Our chosen summary statistic throws away this information by computing the maximum absolute deviation (MAD) over all *t* in order to determine whether that value deviates significantly from the spatially random case. However it may be of interest to determine whether clustering or repulsion between spines occured at specific inter-point distances *t*. Ang’s correction normalizes the K-function such that the theoretical *K*(*t*) = *t* for all *t*, so we can easily use this as a reference point. Figure 7 shows the K-function for the same 9 example dendrites used for the Q-Q plots of Figure 5. Each graph shows the observed $\hat{K}(t)$ function (black), the theoretical *K*(*t*) function (red) as well as the two-sided 5% and 95% point-wise simulation envelopes as a function of the radius *t*. Following the description of the point-wise simulation envelope above we calculated these lower and upper envelopes at the 5% and 95% percentiles per t-value in the interest of checking if *any* t-value fell outside of this range. Since the black curves do not leave the gray shaded area for any value of t, the deviation from spatially random was insignificant at the 10% level for every t-value and is in agreement with our previous conclusion using the MAD statistic. This observation holds for almost all of the 485 dendrites we inspected visually, with no specific t-value evidencing either repulsion or clustering.

## Conclusions

The models used in this work allow spatial prediction of spine types, which has not previously been studied. The conclusions presented here relate to qualities of neurons in dissociated culture. We acknowledge that some of these results will most likely not hold for in vivo settings due to neuronal interactions not modeled here, but maintain that the statistical methods used here will be useful and easily applicable. Specifically, we found here that spine type and density are not dependent on the distance from the cell body, and these observations are likely to change for in vitro slices or micro-injection of fixed brain tissue.

We also note that we chose not to deconvolve our data because of its high contrast. We acknowledge that this choice may have precluded the image analysis software from detecting some stubby spines among the halo of the bright dendrites, but we do not feel this significantly impacted our results. As a partial compensation for this effect we used NeuronStudio’s in-built automatic z-smear compensation, and for more details on this we refer the reader to [22,23].

Although in this study the spine distributions seemed to be completely spatially random it is possible that we will find studies using different neuronal types and treatments where this is not true. In these cases, where spine density may vary with distance from the cell body, it would be interesting to test for inhomogeneous patterns of points such as the hard core Strauss Process used in [43]. We could also place an exponentially decaying function to model the interaction between spine types within a certain radius or experiment with other pairwise interaction functions such as those used by Diggle, Gates and Stibbard [44] or Diggle and Gratton in [45].

We find it an interesting result that spines were not spatially clustered when type was disregarded, as shown by the linear network K-function analysis, however spine types do tend to group together as shown by the MLR analysis. We would like to note that these results are not contradictory because they are in fact measuring different quantities. The MLR results tells us that, regardless of their densities along the dendrite, if we have a spine which is of a given type, its 3 nearest neighbors are likely to be of the same type. The K-function, on the other hand, tells us that regardless of type the spines’ locations along the dendritic network are spatially random. These two results provide complementary information and together could aid us in future modeling tasks such as simulation of neuronal growth. For example, we could first place spines uniformly along the dendritic network, and then decide the types of those spines based on the type of information given by the MLR model. As future work we plan to analyze the network cross K-function [15] of the dendritic network, which models the spine distribution as a multi-type point process and therefore provides information about repulsion and clustering of each spine type with each other spine type, modeling both density and type simultaneously.

Generally previous studies such as [46-49] have relied on physiology or biochemical markers to validate their neuronal properties. The quantitative morphological features described here provide an additional phenotypic dimension for these analyses. Likewise these approaches can be applied to phenotypic analyses of neuronal cultures following over-expression or suppression of specific genes to capture their effect on a complex phenotype. As mentioned in the Introduction section, the only other study we are aware of which analyzes clustering of dendritic spines in monkey brains is [14]. The authors of this work study the number of "clustered spines" on each dendritic segment, where a cluster is defined as a group of 3 or more spines. The method used here defines clustering as a statistically significant positive deviation in the linear K-function from the theoretical value of the spatially random linear K-function. We believe our method to be more principled and our results easier to interpret than those of [14] due to the more formal statistical definition of clustering.

We chose to use dissociated hippocampal cultures because they are widely used and they allow us to perform an in-depth and automated analysis with larger spine populations than most previous studies. These approaches will be important in assessing features of neurons derived from human induced pluripotent stem cells which have so far not been characterized by detailed morphological features. Our paper utilized a highly simplified neuronal culture system to develop the statistical and computational tools for more advanced in vivo studies needed to address the aforementioned bigger biological questions. Our overall hypothesis was that we can utilize imaging and statistical analyses to capture features of spine distributions that can be used for testing hypotheses in in-vivo settings. Indeed, we have been conservative about hypotheses and findings concerning spine type clustering because any conclusions we might reach on the specifics of spine distribution would be limited to the neuronal culture system we studied.

## Availability of supporting data

All the image stacks and NeuronStudio annotation files supporting the results of this article are available in the BISQUE repository, http://bisque.ece.ucsb.edu/client_service/view?resource=http://bisque.ece.ucsb.edu/data_service/dataset/2653471.

## Abbreviations

DIV: Day in vitro; LLM: Log-linear model; SD: Distance from the soma to the spine along the centerline of the dendrite; BO: Branch order of the dendritic segment on which a spine lies; N1, N2, N3: The spine types of the 1st, 2nd, and 3rd nearest neighbors along the dendrite, respectively; MLR: Multinomial logistic regression; CSR: Completely spatially random; FDR: False discovery rate; MAD: Maximum absolute deviation.

## Competing interests

The authors declare that they have no competing interests.

## Authors’ contributions

AJ designed the study, wrote the software to perform the statistical analyses, interpreted the results and drafted the manuscript. SB cultured and collected the data and drafted the section on Cell Imaging. KK conceived of the study and helped to draft the manuscript. BM coordinated the effort between departments and reviewed the manuscript. All authors read and approved the final manuscript.

## Supplementary Material

Additional file 1 **Table of 4-way LLM coefficients.** This table shows the 4-way interaction LLM coefficients which are significant at the 0.1*%* level. Note that only one interaction between type and either branch order or soma distance (highlighted in green) is significant in the entire table. This further proves the result that these interactions are not very important to the overall model of frequencies. Click here for file

Additional file 2 **AIC Stepwise models for 3-way LLM.** This table shows the results of the AIC stepwise algorithm using an LLM with up to 3-way interactions. The models arrived at by this method are shown in the caption above the table. From this table we can see that if we do allow 3rd order interactions, the strongest 3rd order correlation over all experiments is that of DIV, SD and BO, which makes sense because all three of these quantities should intuitively increase together. Click here for file
