# Supplementary material for: Statistical analysis of dendritic spine distributions in rat hippocampal cultures
Source: BMC Bioinformatics. 2013 Oct 2;14:287. doi: 10.1186/1471-2105-14-287 (PMC3871014; doi:10.1186/1471-2105-14-287)
Supplement: Additional file 1 — Table of 4-way LLM coefficients. This table shows the 4-way interaction LLM coefficients which are significant at the 0.1% level. Note that only one interaction between type and either branch order or soma distance (highlighted in green) is significant in the entire table. This further proves the result that these interactions are not very important to the overall model of frequencies. [file 1471-2105-14-287-S1.pdf]

Table A1. This table shows the 4-way interaction LLM coefficients which are significant at the 0.1% level. Note that only one interaction between type and either branch order or soma distance (highlighted in green) is significant in the entire table.

	Estimate	Std.Error	z-value	Pr(> $|z|$)
Experiment 1				
(Intercept)	4.585e+00	1.010e-01	45.389	< 2e-16 ***
div21	5.683e-01	1.264e-01	4.495	6.95e-06 ***
typestubby	-5.960e-01	1.695e-01	-3.517	0.000437 ***
typethin	-1.289e+00	2.174e-01	-5.931	3.01e-09 ***
bo3	-8.714e-01	1.860e-01	-4.685	2.80e-06 ***
bo4	-2.187e+00	3.180e-01	-6.878	6.07e-12 ***
bo5	-3.892e+00	7.143e-01	-5.449	5.08e-08 ***
sd2	-1.253e+00	2.143e-01	-5.846	5.03e-09 ***
sd3	-1.589e+00	2.454e-01	-6.477	9.36e-11 ***
div7:typethin	8.992e-01	2.703e-01	3.327	0.000878 ***
typestubby:bo3	9.599e-01	2.647e-01	3.627	0.000287 ***
bo3:sd2	2.124e+00	2.837e-01	7.486	7.10e-14 ***
bo4:sd2	3.249e+00	3.862e-01	8.413	< 2e-16 ***
bo5:sd2	4.703e+00	7.495e-01	6.274	3.51e-10 ***
bo3:sd3	2.065e+00	3.158e-01	6.539	6.18e-11 ***
bo4:sd3	3.509e+00	4.055e-01	8.653	< 2e-16 ***
bo5:sd3	6.002e+00	7.525e-01	7.976	1.51e-15 ***
Experiment 2				
(Intercept)	5.094e+00	7.833e-02	65.033	< 2e-16 ***
div7	-6.511e-01	1.338e-01	-4.867	1.14e-06 ***
typethin	3.573e-01	1.021e-01	3.499	0.000467 ***
bo4	-1.405e+00	1.765e-01	-7.962	1.70e-15 ***
bo5	-1.483e+00	1.821e-01	-8.143	3.86e-16 ***
sd2	-1.380e+00	1.747e-01	-7.900	2.80e-15 ***
sd3	-2.609e+00	2.991e-01	-8.722	< 2e-16 ***
sd4	-2.897e+00	3.424e-01	-8.459	< 2e-16 ***
div21:bo3	-1.256e+00	2.071e-01	-6.068	1.30e-09 ***
div7:bo3	-8.222e-01	2.348e-01	-3.501	0.000463 ***
bo2:sd2	8.531e-01	2.059e-01	4.143	3.42e-05 ***
bo3:sd2	1.657e+00	2.067e-01	8.018	1.08e-15 ***
bo4:sd2	3.005e+00	2.459e-01	12.220	< 2e-16 ***
bo5:sd2	2.917e+00	2.517e-01	11.587	< 2e-16 ***
bo2:sd3	1.327e+00	3.313e-01	4.006	6.18e-05 ***
bo3:sd3	2.128e+00	3.281e-01	6.485	8.88e-11 ***
bo4:sd3	4.090e+00	3.466e-01	11.801	< 2e-16 ***
bo5:sd3	4.982e+00	3.450e-01	14.441	< 2e-16 ***
bo3:sd4	1.777e+00	3.814e-01	4.659	3.18e-06 ***
bo4:sd4	3.551e+00	3.940e-01	9.014	< 2e-16 ***
bo5:sd4	5.590e+00	3.822e-01	14.625	< 2e-16 ***
Experiment 3				
(Intercept)	3.689e+00	1.581e-01	23.331	< 2e-16 ***
sd3	-1.743e+00	4.097e-01	-4.254	2.10e-05 ***
sd4	-2.079e+00	4.743e-01	-4.384	1.17e-05 ***
bo5:sd2	2.015e+00	4.073e-01	4.946	7.56e-07 ***
bo3:sd3	1.725e+00	4.508e-01	3.827	0.000130 ***
bo4:sd3	2.182e+00	4.739e-01	4.605	4.12e-06 ***
bo5:sd3	2.902e+00	5.000e-01	5.805	6.44e-09 ***
bo5:sd4	3.818e+00	5.463e-01	6.988	2.79e-12 ***
