# Supplementary material for: Statistical analysis of dendritic spine distributions in rat hippocampal cultures
Source: BMC Bioinformatics. 2013 Oct 2;14:287. doi: 10.1186/1471-2105-14-287 (PMC3871014; doi:10.1186/1471-2105-14-287)
Supplement: Additional file 2 — AIC Stepwise models for 3-way LLM. This table shows the results of the AIC stepwise algorithm using an LLM with up to 3-way interactions. The models arrived at by this method are shown in the caption above the table. From this table we can see that if we do allow 3rd order interactions, the strongest 3rd order correlation over all experiments is that of DIV, SD and BO, which makes sense because all three of these quantities should intuitively increase together. [file 1471-2105-14-287-S2.pdf]

Table A2. AIC Stepwise-fit models of LLM with up to 3-way interactions
EXP 1 Stepwise Final Model:
freq   div + type + bo + sd + bo:sd + div:bo + div:type + div:sd + type:bo + type:sd + div:bo:sd + div:type:bo, AIC=1243.92
EXP 2 Stepwise Final Model:
freq   div + type + bo + sd + bo:sd + div:bo + div:sd + div:type + div:bo:sd, AIC=927.75
EXP 3 Stepwise Final Model:
div + type + bo + sd + bo:sd + div:sd + div:type + div:bo + type:sd + type:bo + div:bo:sd + div:type:sd + div:type:bo, AIC=1165.38

	Df	Deviance	AIC
Experiment 1			
none		137.31	1243.9
- type:sd	6	154.00	1248.6
+ div:type:sd	12	118.35	1249.0
+ type:bo:sd	24	96.12	1250.7
- div:type:bo	16	191.02	1265.6
- div:bo:sd	24	478.78	1537.4
Experiment 2			
none		106.83	927.75
+ type:sd	6	97.94	930.87
+ type:bo	8	102.10	939.03
- div:type	4	247.02	1059.95
- div:bo:sd	24	470.21	1243.13
Experiment 3			
none		102.32	1165.4
- div:type:bo	16	134.54	1165.6
+ type:bo:sd	24	68.79	1179.8
- div:type:sd	12	147.51	1186.6
- div:bo:sd	4	398.35	1413.4
